# Effect of the supplementation of exogenous complex non-starch polysaccharidases on the growth performance, rumen fermentation and microflora of fattening sheep

**DOI:** 10.3389/fvets.2024.1396993

**Published:** 2024-05-16

**Authors:** Yuyang Xue, Haobin Sun, Hongyong Guo, Cunxi Nie, Shanshan Nan, Qicheng Lu, Cheng Chen, Wenju Zhang

**Affiliations:** ^1^College of Animal Science and Technology, Shihezi University, Shihezi, China; ^2^Xinjiang Tianshan Junken Animal Husbandry Co., Ltd., Shihezi, China

**Keywords:** non-starch polysaccharidases, rumen fermentation, microbiome, sheep, enzyme

## Abstract

The objective of this study was to evaluate the effects of exogenous non-starch polysaccharidases (a mixture of cellulase, xylanase, β-glucanase and mannanase) on the growth performance and nutrient digestibility, rumen fermentation, and rumen microflora of sheep. The animal trial was conducted using 36 5-month-old female fattening hybrid sheep (Duolang♂ × Hu♀) who were randomly assigned into four groups comprising nine sheep per treatment: CON, T1, T2, and T3, with 0, 0.1, 0.3, and 0.5% NSPases/kg DM of TMR, respectively. This complex enzyme product was screened for optimal ratios based on previous *in vitro* tests and responded positively to the *in vitro* fermentation of the TMR. When treated with NSPases, there was a non-linear effect of average daily gain and feed conversion rate, with the greatest improvement observed in the T2 group. There were no significant differences (*p* > 0.05) in nutrient intake or apparent digestibility among the NSPase-supplemented groups. In addition, T2 group had a significantly higher acetate to propionate ratio and pH (*p* < 0.05) than the other groups, and NH_3_-N and microbial protein concentrations showed a quadratic curve. The results revealed that both immunoglobulins and serum hormones increased linearly with addition (*p* < 0.05). As the T2 group showed the best growth performance, the CON and T2 groups were subjected to rumen metagenomic analysis. The results showed higher abundance of bacteria and lower abundance of Viruses in the rumen microbiota of the T2 group compared to the CON group. In addition, Uroviricota and Proteobacteria abundance was significantly lower in the T2 group than in the CON group at the phylum level (*p* < 0.05). These results suggest that the supplementation of high-concentrate rations with NSPases enhance immunity, reduces virus abundance in the rumen, improves rumen health, and promotes rumen fermentation. Our findings provide novel insights for improving growth performance and alleviating inflammatory responses arising from high concentrate feeding patterns in ruminants. However, the biological mechanisms cannot be elucidated by exploring the composition of rumen microbe alone, and further studies are required.

## 1 Introduction

In recent years, the application of enzymes in ruminants to improve feed utilization and optimize growth performance has been identified as a valuable measure. Non-starch polysaccharidases (NSPases) have been increasingly investigated as green and harmless additives for the degradation of non-starch polysaccharides (1–3). Previous studies have shown that the main effects of enzyme supplementation in ruminant diets are: breaking chemical bonds in the diet; improving the availability of nutrients in the digestive system; and increasing total enzyme activity in the rumen (4, 5). A meta-analysis showed inconsistent effects of added enzyme preparations (cellulase, xylanase, glucanase, ferulose esterase and combinations of each of their single enzymes) (6), and the reasons for their inconsistent results may be due to the source of enzymes, the amount of enzymes added, the experimental animals and the different diets on which enzymes acted in each study. Such as: Valdes reported that the supplementation of the diet of Suffolk lambs with enzyme products containing cellulases, xylanases, proteases and α-amylases improved average daily gain and feed conversion rate (7). Sakita fed an fibrolytic enzyme product to growing lambs which increased their dietary digestible energy and ADF digestibility (8). Similar results were reported by Shi et al. (9). Moreover, numerous studies have shown that supplementing dairy cow diets with enzymes can improve lactation performance (10–12). In contrast, Peters fed a combination of xylanase and dextranase to lactating cows and found no improvement in milk production (13). And Vinyard exported exogenous enzymes did not improve *in vitro* ruminal fermentation in the diet of high-producing dairy cows (14). Although there are many reports on combination enzymes, there are no specific standards or bases for the application of combination enzymes and the amount to be added.

Non-starch polysaccharides refer to the cellulose, xylan, β-glucan, xyloglucan, galactomannan and pectin present in plant cell walls which are difficult to degrade. The main basal polysaccharides in the plant cell wall other than cellulose are arabinoxylan and (1,3)(1,4)-β-D-glucan (15). (1,3)(1,4)-β-D-glucan binds cellulose and other polysaccharides tightly and forms the main part of the endosperm wall (16). The rumen of ruminants acts as a natural reactor for the degradation of plant cell walls, which includes most fiber-degrading bacteria such as Ruminococcus, Clostridium, and Prevotella (17). These microbes degrade non-starch polysaccharides and then generate volatile fatty acids (VFAs). NSPases include cellulases, xylanases, β-glucanases, and mannanases, which can synergize with rumen microbes, break the cell wall and promote the degradation of the rations. Cellulases and xylanases are the most widely used exogenous enzymes in animals and play important roles in nutrient utilization. In addition, enzyme supplementation has a potential effect on the composition and metabolic capacity of rumen microbes (18, 19), which can alter the composition of microbial communities (20, 21) and improve rumen fermentation and health (22). In the actual production process, most farmers use a sloppy meat farming model to obtain higher economic benefits. Particularly in the late fattening stage, fast short-term fattening of sheep meat is achieved by increasing the proportion of concentrate in the diet. However, feeding a high proportion of concentrate accelerates rumen fermentation and produces large amounts of volatile fatty acids, which cause acidosis, inflammatory reactions and nutritional metabolism (23). Currently, various additives, including enzymes, are used to attenuate the negative effects of high concentrations in ruminants fed high-concentrate diets. Studies on the supplementation of high concentrate diets with complex NSPases in sheep have not yet been conducted.

The effect of sex has been shown to be a major determinant of differences in growth and deposition rates across body tissues and carcasses (24). Regarding feed efficiency, it is well known that males have a better ability to convert feed into weight gain and carcass component tissues than females (25). Males showed a more pronounced rate of carcass gain, deposited more carcasses and gained more weight per kg of DM consumed than females (*p* < 0.05), with castrated animals in an intermediate position. This is consistent with the effects of sex on growth, body composition and carcass of animals reported by Washaya (26) and Paulino et al. (26). Purwin et al. also showed that sex has a significant effect on the rate of change in body weight and relative growth rate (27). Most of the current studies have focused on males, while only few studies have been conducted on female meat ruminants; therefore there is a need to study females to improve production performance. We hypothesized that supplementation with exogenous NSPases could improve rumen fermentation and production performance in ewes.

We hypothesized that a specific combination of NSPases (i.e., cellulose, xylanase and mannanases) applied to a high-concentrate diet would break the cell wall and, improve rumen fermentation and the growth performance of sheep. This study evaluated the effects of NSPases on the intake and total apparent digestibility of nutrients, rumen fermentation, and rumen microbial composition in sheep.

## 2 Materials and methods

### 2.1 Preparation of the enzyme product

NSPase products were purchased from Shandong Longkot Enzyme Preparation Co., Ltd. (Linxi, Shandong, China). Four commercial enzyme preparations were utilized: cellulase originating from Trichoderma longibrachiatum, xylanase originating from Aspergillus oryzae, β-glucanase originating from Trichoderma longibrachiatum, and mannanase originating from *Bacillus lentus*. In this assay, enzyme activity was defined as the amount of enzyme that release 1 μmoL of the reducing sugar from 1 mg/mL solution per minute at 39°C, pH 6.5, which was defined as 1 enzyme activity unit (U).

According to the results of previous studies, the enzyme activities of cellulase, xylanase, β-glucanase and mannanase were 18,000, 76,000, 100,000, 61,000 and 30,000 U/mL, respectively at 39°C, pH 6.5 (consistent with rumen). According to the sample diets and *in vitro* studies, the optimal ratio of cellulase: xylanase: β-glucanase: mannanase is = 1,000: 250: 500: 50 (U/g, dry matter basis). Considering the differences between the *in vivo* and *in vitro* conditions, this feeding trial was conducted by rationalizing the complex NSPases screened *in vitro* and adding them according to the daily intake of the test sheep.

### 2.2 Animals, diets and experimental design

A total of 36 female Duolang (♂) × Hu (♀) sheep aged 5 months with an initial live body weight of 35.3 ± 2.4 kg were divided into four treatment groups. All test sheep were housed individually in pens with feed and water provided *ad libitum*. The experimental time lasted 6 weeks, with the first weeks for adaptation. Based on the amount of complex enzyme added, the samples were divided into the control group CON (0%), test group T1 (0.1%), medium dose additive treatment group T2 (0.3%), and high dose additive treatment group T3 (0.5%). Before the morning feed, the NSPases were diluted in deionized water and sprayed on the TMR using a handheld sprayer. As a control, a similar amount of deionized water without NSPases was sprayed. All sheep were provided water *ad libitum* and a total mixed ration (Table 1) twice daily at 08:00 and 18: 00 h. The diet was designed according to the feeding standards for meat-producing sheep (NY/T 816-2021, Ministry of Agriculture, China). The detailed ingredients and nutritional compositions of the mixed rations are listed in Table 1.

**Table 1:** Feed ingredients and chemical composition of basal total mixed ration on a dry matter (DM) basis.

Items	Content
Ingredient, % of DM	
Corn	49.40
Soybean meal	17.70
Wheat bran	11.60
Cottonseed meal	6.30
Alfalfa meal	5.00
Corn straw	5.00
Corn silage	2.00
CaHCO_3_	1.00
Premix^1^	1.00
NaCl	0.50
Limestone	0.50
Chemical composition of diet^2^	
DM (% as fed)	85.25
DE (MJ/kg)	12.40
CP (% DM)	18.94
NDF (% DM)	19.21
ADF (% DM)	10.26
Ca (% DM)	0.66
P (% DM)	0.44

1 Premix was provided per kg of diet: I 0.85 mg, Fe 53 mg, Cu 12 mg, Mo 0.7 mg, Co 0.4 mg, Mn 43 mg, Zn 35 mg, Se 0.3 mg, VA 940 IU, VD 132 IU, VE 12.8 IU.

2 Ca, P, and DE was calculated value, the rest were measured values.

### 2.3 Growth performance

On day 0 of the pre-feeding period and day 35 of the formal period, the body weight of each sheep was measured before the morning feeding to obtain the initial and final weights and calculate the average daily weight gain. Feed intake and refusal were recorded daily for each replicate group to calculate DM intake and feed conversion rate.

### 2.4 Apparent digestibility of nutrients

Feed and fecal samples were collected on days 30 and 35 of the experiment. Six sheep were randomly selected in each group, with a total of six sheep in each group and fecal samples was collected using the rectal end collection method. Samples from each sheep were mixed for 5 d. All feed and fecal samples were dried for 48 h in a 65°C oven before being moistened for 24 h. They were crushed through a 1 mm screen, sealed in a 150 × 220 mm sealed bag and stored at 4°C for nutritional component analysis. Based on the acid insoluble ash (AIA) method described previously (28), the digestibility of ADF, NDF, and CP in the feed and fecal samples was evaluated. To assess the DM concentration, samples were dried to a consistent weight at 65°C. An automatic fiber analyzer, a Kay nitrogen analyzer, and a Soxhlet extraction system were used to determine ADF, NDF, and CP, respectively. The AIA approach was employed using the following formula:


$$Apparentdigestibility\;\left(\%\right)=100-\left[\left(M_{\mathrm{2n}}\times M_{\mathrm{1m}}\right)/\left(M_{\mathrm{1n}}\times M_{\mathrm{2m}}\right)\right]$$

where M_1m_ is the AIA content in the diet (%), M_2m_ is the AIA content in the fece, M_1n_ is the nutrient content in the diet (%), and M_2n_ is the nutrient content in the fece.

The calculation formula of feed conversion ratio (FCR) is as follows:


FCR(kg/kg)=Total feed intake/weight gain

### 2.5 Blood collection and analyses

Approximately 10 mL of blood was collected from the jugular vein of each sheep using a sterile transfusion needle and negative-pressure blood aspiration tube. Serum was obtained via centrifugation at 3,500 r/min for 10 min and stored at −20°C. Serum hormone (growth hormone, insulin-like growth factor-1, and leptin) and immune (IgA, IgG, and IgM) concentrations were determined according to the manufacturer’s instructions using enzyme-linked immunosorbent assay kits (Nanjing Jiancheng Biotechnology Co., Nanjing, China).

### 2.6 Rumen fermentation parameters

Ruminal fluid samples were collected from all sheep after 2 h of morning feed by an oral stomach tube (1.2 m, 6.0 mm) with negative pressure. The first liquid sample was discarded to reduce saliva pollution. Then, the filtered rumen fluid samples were divided into two parts, one in 50 mL centrifuge tubes and stored at −20°C for NH_3_-N, MCP, and VFA determination and the other in 5 mL cryo-storage tubes and stored at −80°C for rumen microbial analysis. According to the colorimetric method described by, NH_3_-N concentrations were determined using a spectrophotometer (721G, Shanghai, China). MCP concentration was measured using the bicinchoninic acid method. The VFA concentrations were determined using chromatography (Agilent GC7890B, United States).

### 2.7 DNA extraction and metagenomic analysis

As supplementation with medium-dose NSPases showed better results, six sheep (the same six sheep from which feces were collected) were selected in the CON and T2 groups, for DNA extraction and metagenomic analysis. Total DNA was extracted from the rumen samples, and the DNA concentration and purity were determined using 1% agarose gel electrophoresis. The sample genes were broken into 400 bp fragments using an ultrasonic fragmentation instrument (Covaris M220, United States), and library preparation was completed by end repair, addition of sequencing junctions, gene purification, and PCR amplification. Sequencing was performed by Majorbio Inc. (Shanghai, China) on an Illumina HiSeq 4,000 platform (Illumina Inc., San Diego, California, United States). The raw data were quality-controlled using the fastp software^1^ to obtain high-quality clean reads by removing low-quality splice sequences, reads, and bases. ORF prediction of contig sequences was performed using Prodigal software (29). IAMOND^2^ and MEGAHIT assembly software were used for the species annotation and sequencing data assembly.

### 2.8 Statistical analysis

All data were subjected to simple polynomial regression using SAS Proc Mixed SAS version 9.4, Statistical Analysis for Windows, Institute Inc., Cary, NC, United States. All data are expressed as mean and SEM. Correlation analysis was performed between rumen microbes, rumen fermentation and serum indicators, and correlation coefficients were calculated based on Spearman correlation distances. Heatmaps were constructed using the Origin Pro 2021 software. The Wilcoxon rank-sum test (two-tailed test) was performed separately to compare the functional characteristics and differentiation of the CAZys between the CON and T2 groups.

## 3 Result

### 3.1 Effect of increasing NSPase supplementation on growth performance and apparent digestibility in fattening sheep

Average daily gain and feed conversion rate in the different treatments showed a quadratic curve correlation, and the T2 group had a higher average daily gain than the T3 group (*p* < 0.05; Table 2). NSPases treatment had no effect on final body weight or DMI (*p* > 0.05; Table 2). The addition of NSPases had no significant effect on the nutrient intake or digestibility of fattening sheep (*p* > 0.05; Table 3).

**Table 2:** Effect of the supplementation of dietary exogenous non-starch polysaccharidases on growth performance and feed efficiency of fattening sheep.

Items	Treatment^1^				SEM	*p*-value^2^		
	CON	T1	T2	T3		Treat	Linear	Quadratic
Initial BW, kg	35.30	34.82	35.34	36.10	0.66	0.938	0.661	0.819
Final BW, kg	42.89	43.01	43.92	42.87	0.71	0.951	0.893	0.916
Average daily gain, g/d	180.69^ab^	194.97^a^	204.23^a^	158.46^b^	6.27	0.048	0.313	0.029
DM intake, kg/d	0.83	0.90	0.94	0.84	0.035	0.717	0.836	0.517
Feed conversion rate	4.62^b^	4.61^b^	4.52^b^	5.46^a^	0.14	0.021	0.043	0.014

1 Different supplementary levels (CON, control group; T1, 0.1% NSPases/kg DMI; T2, 0.3% NSPases/kg DMI; T3, 0.5% NSPases/kg DMI).

2 Treat, Linear and Quadratic represent treat, linear, and quadratic effects, respectively of NSPase addition.

^a,b^Values within a row with different superscripts differ significantly at *p* < 0.05.

**Table 3:** Effect of the supplementation of dietary exogenous non-starch polysaccharidases on apparent digestibility of fattening sheep.

Items^1^	Treatment^2^				SEM	*p*-value^3^		
	CON	T1	T2	T3		Treat	Linear	Quadratic
Intake, (g/d)								
DM	831.83	897.83	939.83	842.94	35.06	0.734	0.823	0.540
OM	792.14	858.84	897.01	801.58	18.89	0.160	0.700	0.090
CP	11.98	12.99	13.57	12.13	0.510	0.717	0.836	0.517
NDF	27.47	29.79	31.11	27.80	1.063	0.610	0.814	0.423
ADF	6.65	7.23	7.55	6.75	0.206	0.401	0.768	0.250
Digestibility, %								
DM	70.30	70.31	70.23	70.34	0.002	0.236	0.810	0.406
OM	77.34	77.19	77.69	77.34	0.090	0.248	0.548.	0.728
CP	64.81	63.97	66.17	65.88	0.624	0.608	0.345	0.631
NDF	66.12	65.58	67.48	66.25	0.389	0.381	0.521	0.744
ADF	44.6	43.6	45.57	47.15	0.979	0.647	0.428	0.460

1 DM, dry matter; OM, organic matter; CP, crude protein; NDF, neutral detergent fiber; ADF, acid detergent fiber.

2 Different supplement levels (CON, control group; T1, 0.1% NSPases/kg DMI; T2, 0.3% NSPases/kg DMI; T3, 0.5% NSPases/kg DMI).

3 Treat, Linear and Quadratic represent treat, linear and quadratic effects, respectively, of NSPases supplementation.

### 3.2 Effect of NSPase supplementation on ruminal fermentation characteristics in fattening sheep

The ruminal pH was significantly lower in the T3 group than in the other groups (*p* < 0.05; Table 4). The NH_3_-N and acetate to propionate ratios in the T2 group were significantly higher than those in the other groups (*p* < 0.05; Table 4).

**Table 4:** Effect of the supplementation of dietary exogenous non-starch polysaccharidases on the ruminal fermentation characteristics of fattening sheep.

Items	Treatment^1^				SEM	*p*-value^2^		
	CON	T1	T2	T3		Treat	Linear	Quadratic
Ruminal pH	6.56^a^	6.55^a^	6.59^a^	6.38^b^	0.007	0.04	0.025	0.007
NH_3_-N, mg/mL	6.96^b^	7.79^b^	10.50^a^	3.87^c^	0.540	<0.01	0.177	<0.01
MCP, mg/mL	153.54^ab^	124.55^b^	150.05^ab^	174.74^a^	7.75	0.148	0.205	0.093
Total VFA, mmol/L	97.05	100.43	96.04	98.40	1.70	0.843	1.000	0.981
Acetate mmol/L	48.07	49.65	50.10	47.67	0.92	0.777	0.906	0.580
Propionate mmol/L	41.13	42.29	37.42	42.88	1.10	0.344	0.919	0.696
Butyrate mmol/L	7.84	7.99	8.52	7.85	0.22	0.717	0.814	0.679
Acetate: Propionate ratio	1.17^b^	1.16^b^	1.37^a^	1.11^b^	0.04	0.067	0.985	0.315

VFA, volatile fatty acid.

1 Different supplementary levels (CON, control group; T1, 0.1% NSPases/kg DMI; T2, 0.3% NSPases/kg DMI; T3, 0.5% NSPases/kg DMI).

2 Treat, Linear and Quadratic represent treat, linear and quadratic effects, respectively, of NSPase supplementation.

^a–c^Values within a row with different superscripts differ significantly at *p* < 0.05.

### 3.3 Effect of NSPase supplementation on serum hormones in fattening sheep

As shown in Table 5, increasing enzyme concentration linearly and quadratically increased the serum concentrations of GH, IGH-1, leptin, IgA, IgG and IgM (*p* < 0.01).

**Table 5:** Effect of the supplementation of dietary exogenous non-starch polysaccharidases on serum hormones of fattening sheep.

Items	Treatment^1^				SEM	*p*-value^2^		
	CON	T1	T2	T3		Treat	Linear	Quadratic
GH	7.88^c^	10.19^b^	11.66^ab^	12.21^a^	0.383	<0.01	<0.01	<0.01
IGH-1	823.94^d^	1066.78^c^	1261.15^b^	1543.32^a^	53.936	<0.01	<0.01	<0.01
Leptin	7.48^c^	8.99^bc^	9.81^b^	11.97^a^	0.379	<0.01	<0.01	<0.01
IgA	179.92^c^	232.16^b^	264.90^a^	284.97^a^	8.385	<0.01	<0.01	<0.01
IgG	47.92^c^	56.28^b^	61.97^ab^	65.91^a^	1.597	<0.01	<0.01	<0.01
IgM	1526.02^b^	2210.20^a^	2273.15^a^	2406.33^a^	69.580	<0.01	<0.01	<0.01

GH, growth hormone; IGF-1, insulin-like growth factor-1.

1 Different supplementary levels (CON, control group; T1, 0.1% NSPases/kg DMI; T2, 0.3% NSPases/kg DMI; T3, 0.5% NSPases/kg DMI).

2 Treat, Linear and Quadratic represent treat, linear, and quadratic effects, respectively, of NSPase supplementation.

^a–c^Values within a row with different superscripts differ significantly at *p* < 0.05.

### 3.4 Effect of NSPase supplementation on rumen microbial diversity and community in fattening sheep

**Figure 1:**
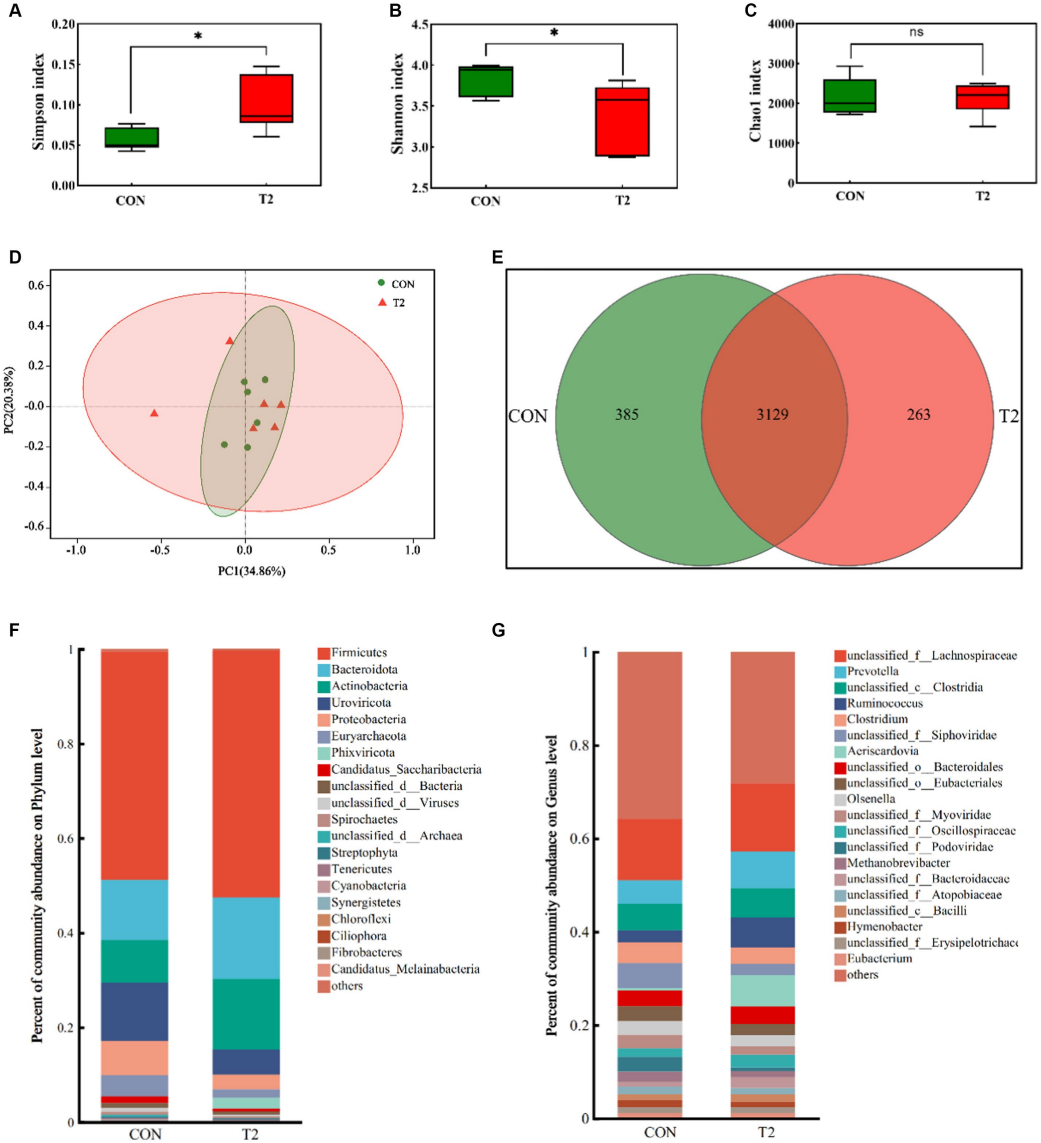
Microbia analysis of the rumen of sheep. **(A–C)** Analysis of alpha diversity of the rumen microbial communities in CON and T2 groups, **(D,E)** Principal coordinates analysis and Venn of the rumen microbial communities in the CON and T2 groups, **(F)** Relative abundance of phylum level in the sheep rumen, **(G)** Relative abundance of genus level in the sheep rumen.

*Bacteria* dominated the rumen microbial communities (>80%), followed by *Viruses* (8–13%), whereas *Eukatyota* (<1%) and *Archaea* (1–5.5%) showed lower abundance (Supplementary Table S1). The Chao1 index did not differ significantly between the CON and T2 groups. The Simpson index of group T2 was significantly higher than that of CON, while the Shannon index of group T2 was significantly lower than that of CON (Figure 1). PCoA plots showed over-lapping clusters in the CON and T2 groups. Among all microbes, *Firmicutes* dominated at the phylum level, followed by *Bacteroidota*, *Actinobacteria, Uroviricota, Proteobacteria,* and *Euryarchaeota* (Figure 1). *Firmicutes, Bacteroidota*, and *Actinobacteria* were higher in the T2 group than in the CON group, *Uroviricota* and *Proteobacteria* were significantly lower than in the CON group (*p* < 0.05; Supplementary Table S2). At the genus level, *unclassified_f_Lachnospiraceae* was the most abundant, followed by *Prevotella*, *unclassified_c_Clostridia*, *Ruminococcus*, *Clostridium*, but none of them were significantly different (Supplementary Table S3). *Unclassified_f_Lachnospiraceae*, *Prevotella*, *unclassified_c_Clostridia*, and *Ruminococcus* were more abundant in the T2 group than in the CON group.

### 3.5 Correlation analysis of apparent digestibility and rumen fermentation with rumen microbial community composition

**Figure 2:**
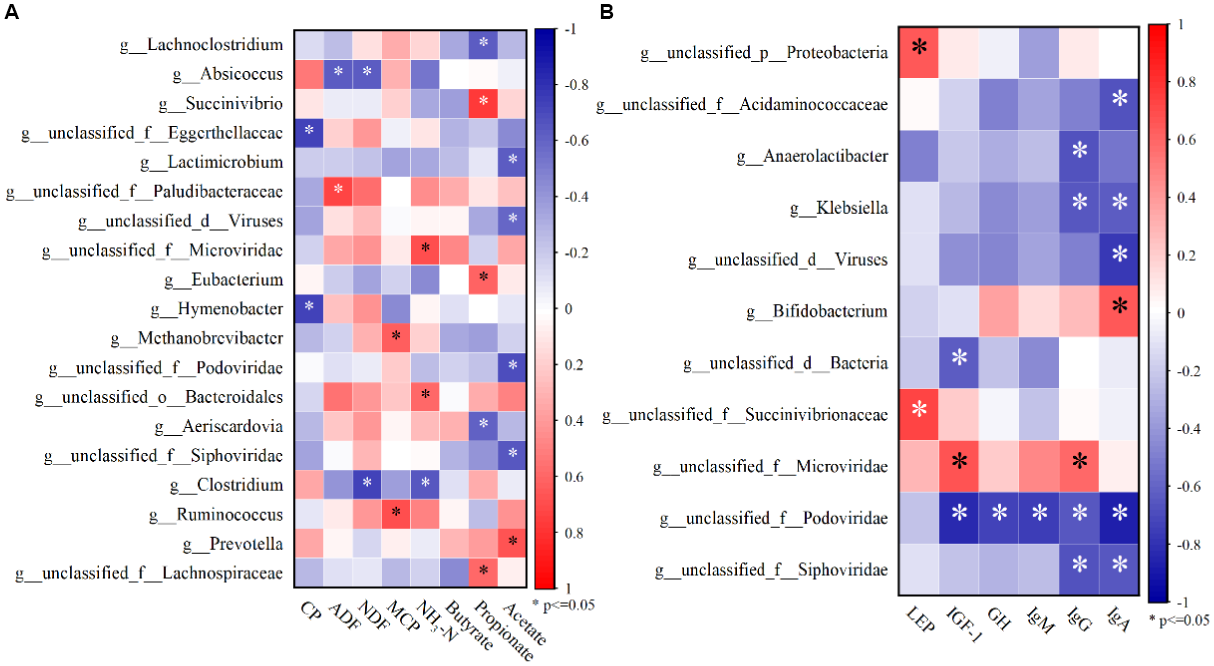
**(A)** Top 50 species with significant correlations with NDF, ADF, CP digestibility and rumen fermentation parameters at rumen microbial genus level abundance; **(B)** Top 50 species with significant correlations with serum hormones and immunoglobulins at rumen microbial genus level abundance.

According to Spearman correlation analysis, ruminal acetate concentrations were negatively correlated with four genera-*Lactimicrobium*, *unclassified_d_Viruses*, *unclassified_f_podoviridae*, and *unclassified_f_Siphoviridae*, and positively correlated with *Prevotella* (*p* < 0.05; Figure 2). Propionate concentrations were negatively correlated with two genera, *Lachnoclostridium* and *Aeriscardovia,* and positively correlated with *Succinivibrio* and *unclassified_f_Lachnospiraceae* (*p* < 0.05; Figure 2). NH_3_-N concentrations were negatively and strongly correlated with *unclassified_f_Microviridae* and *unclassified_o_Bacteroidales* (*p* < 0.05). The MCP concentration was negatively correlated with *Methanobrcvibacter* and *Ruminococus* (*p* < 0.05). NDF and ADF digestibility were negatively and strongly correlated with *Absicoccus*, whereas CP digestibility was negatively correlated with *unclassified_f_Eggerthellaceae* and *Hymenobacter* (*p* < 0.05).

### 3.6 Correlation analysis of serum hormones and serum immunity with rumen microbial community composition

IgA and IgG concentrations were negatively correlated with three genera-*unclassified_f_Siphoviridae*、*nclassified_f_Podovirida*e and *Klebsiella* (*p* < 0.05). IgA concentration was negatively correlated with *unclassified_d_Viruses* and *unclassified_f_Acidaminococcaceae* (*p* < 0.05). IgG concentration was negatively correlated with *Anaerolactibacter* (*p* < 0.05). IgA, IgG, IgM, GH, and IGF-1 concentrations were negatively correlated with *unclassified_f_Podoviridae* (*p* < 0.05). LEP concentration was positively correlated with *unclassified_f_Succinivibrionaccae* and *unclassified_p_Protebacteria* (*p* < 0.05).

### 3.7 Effect of NSPase supplementation on rumen function features in fattening sheep

**Figure 3:**
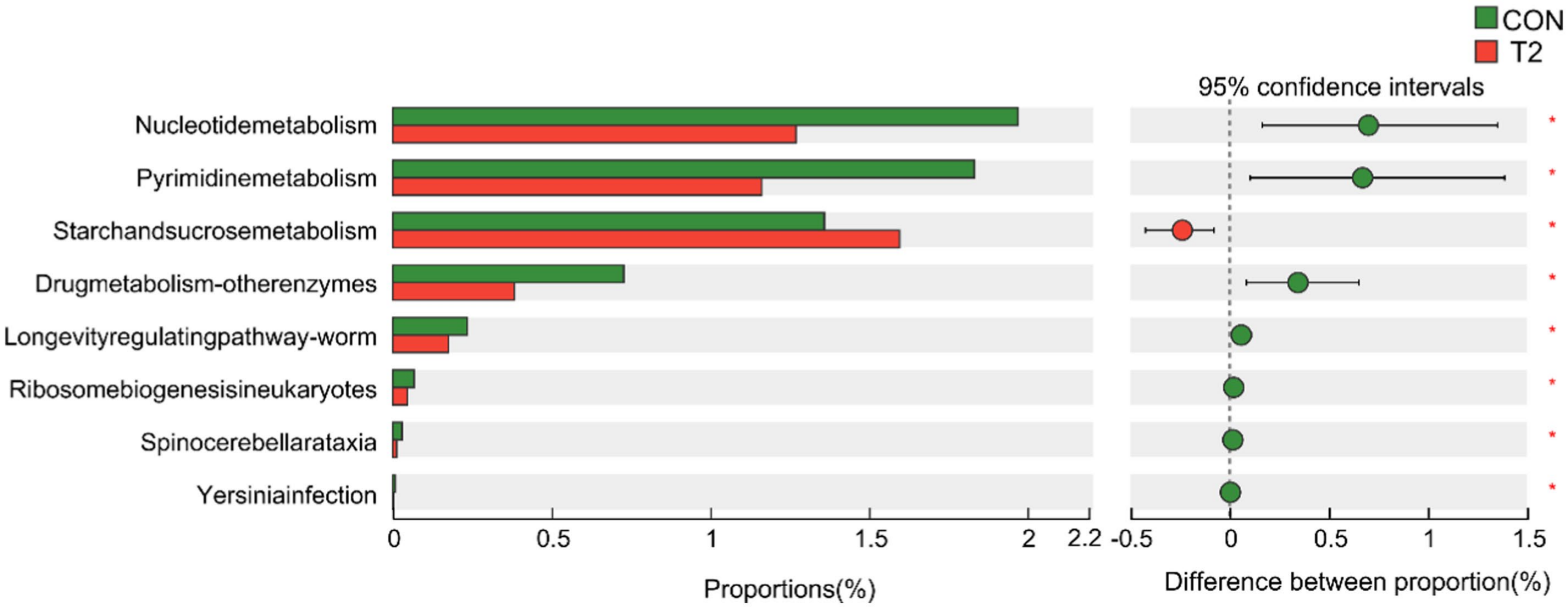
Differential analysis of KEGG Level3 between CON and T2 rumen samples in sheep. Wilcoxon rank-sum test for KEGG pathways. “*” represents *p* < 0.05. CON, control group; T2 = 0.3% NSPases/kg DMI.

**Figure 4:**
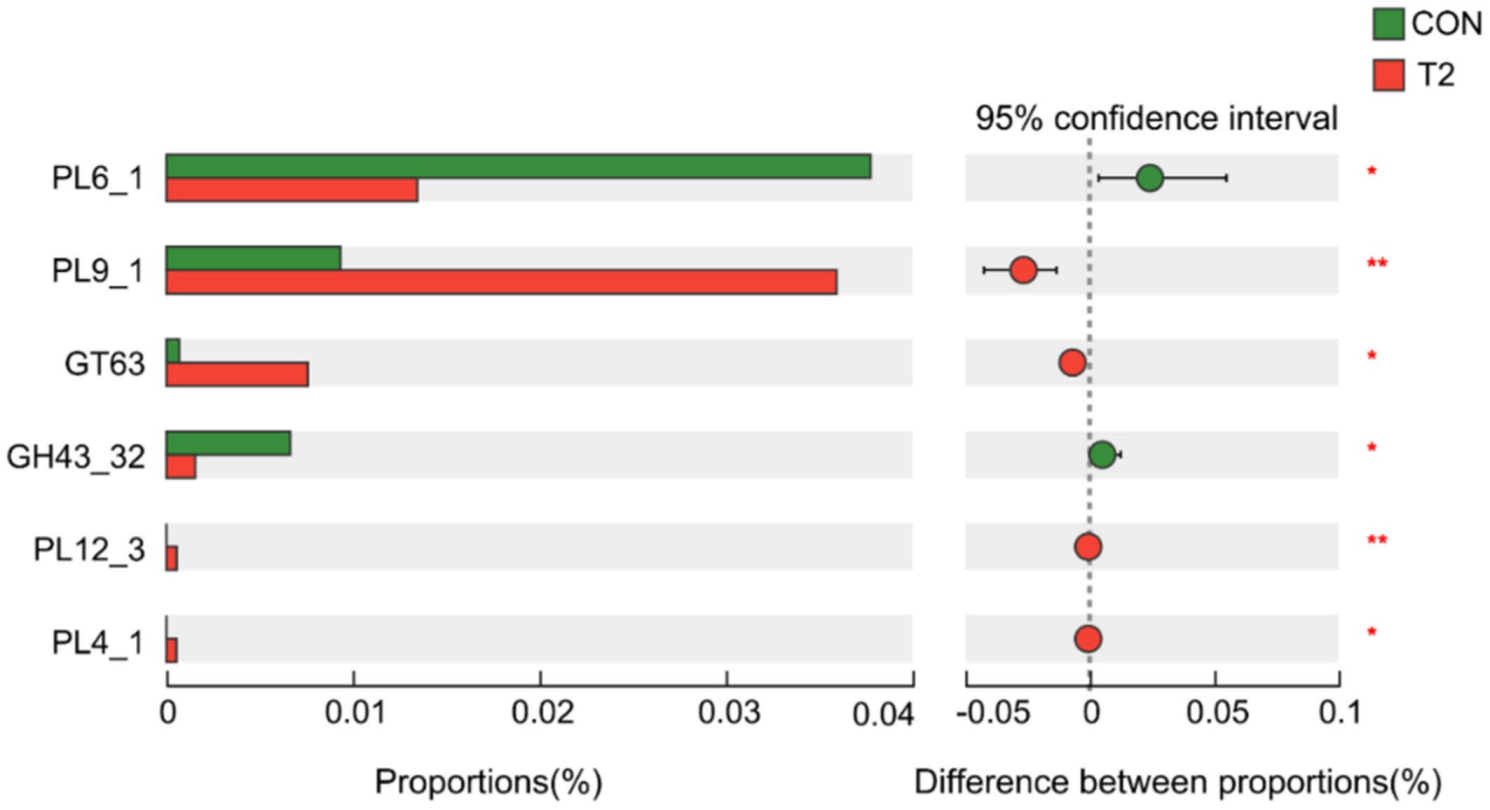
Distinction analysis of CAZy in the rumen samples of sheep in the CON and T2 groups using Wilcoxon rank-sum test. “*” represents *p* < 0.05, and “**” represents *p* < 0.01. CON, control group; T2 = 0.3% NSPases/kg DMI.

A Wilcoxon rank sum test of KEGG Level3 levels in the CON and T2 groups showed that microbes in the T2 group were mainly enriched in starch and sucrose metabolism pathways, whereas microbes in the CON group were mainly enriched in pathways related to nucleotide metabolism, pyrimidine metabolism, drug metabolism-other enzymes, longevity-regulating pathway-worm, ribosome biogenesis in eukaryotes, spinocerebellar ataxia, and functional properties of Yesinia infection (Figure 3). Based on KEGG annotation, the differences in KO and Enzymes involved in starch and sucrose metabolism pathways were analyzed between the two groups, and no significant species differences were found in KO and enzymes of both CON and T2 groups. In addition, the abundance of CAZy was analyzed differently in the CON and T2 groups. The results showed that the T2 group showed high abundance of PL9_1 (pectate lyase), GT63 (DNA β-glucosyltransferase), PL12_3 (heparin lyase), PL4_1 (rhamnogalacturonan lyase), but PL6_1 (alginate lyase) and GH43_32 (β-D-galactofuranosidase) were lower than the CON group (Figure 4).

### 3.8 Correlation analysis between NSPase supplementation and rumen fermentation characteristics

**Figure 5:**
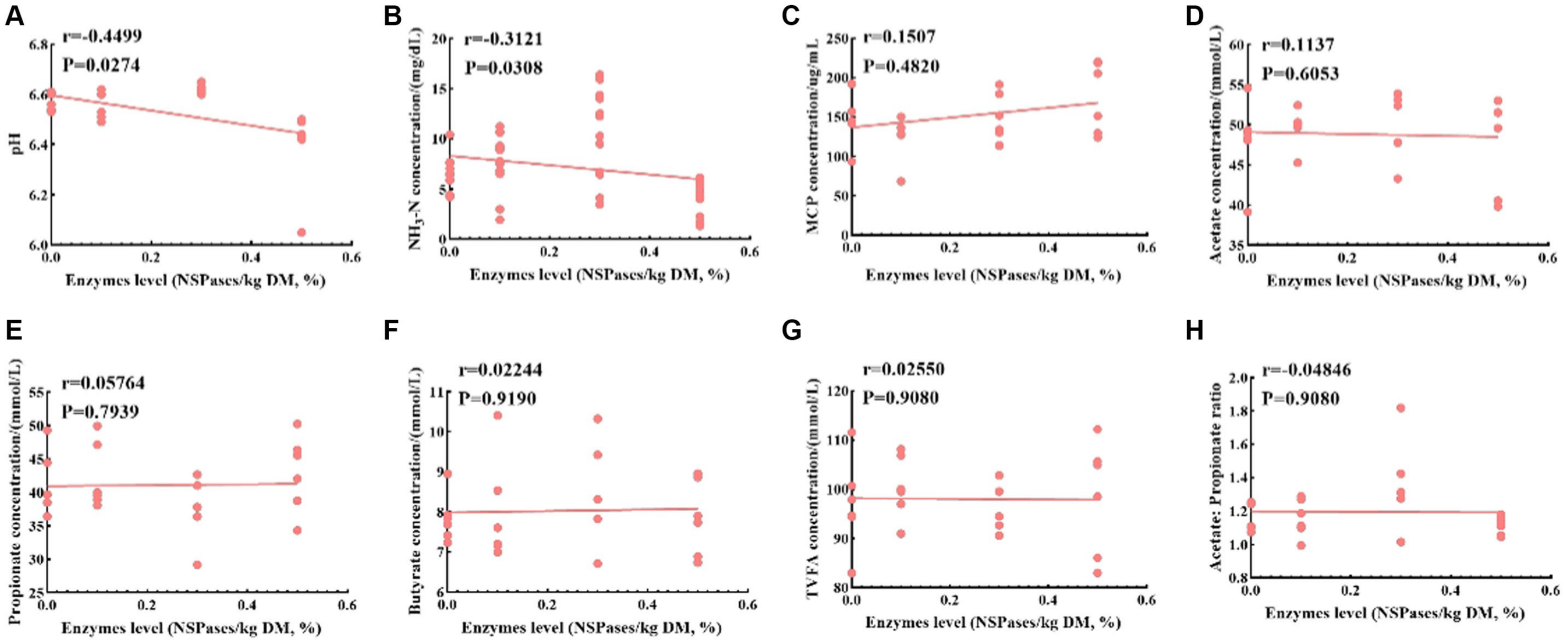
Spearman’s correlation analysis between enzyme addition levels and rumen fermentation characteristics. **(A-H)** represent the correlation analysis of different enzyme additions with pH, NH3-N, MCP, Acetate, Propionate, Butyrate, Acetate: Propionate ratio, respectively. *r*-value: relative coefficient, *r* >0 indicates positive correlation, *r* >0 indicates negative correlation; *p*-value: significance, *p* <0.05 indicates significant correlation, *p* >0.05 indicates non-significant correlation.

Different additions of exogenous complex NSPases were subjected to Spearman’s correlation analysis with rumen fermentation characteristics, as shown in Figure 5; pH and NH_3_-N were significantly negatively correlated with enzyme addition (*p* < 0.05), A/P was negatively correlated with enzyme addition, and MCP, acetate, propionate, butyrate and TVFA were positively correlated with enzyme addition.

## 4 Discussion

Animal growth requires an optimal amount of feed, while simultaneously, improving the digestibility of feed is essential to maximize the quality of growth. Growing animals may exhibit different metabolic and digestive disorders that affect their performance and health. In this study, we aimed to improve the growth performance and health of fattening sheep by adding an exogenous complex of NSPases. It has been hypothesized that this complex enzyme primarily improves fiber digestion and maintains increased microbial activity by interacting with endogenous rumen enzymes. Positive effects on animal health and metabolism have been reported.

Supplementation with NSPases in this study had no significant effect on the digestibility of DM, OM, CP, ADF, and NDF in any of the groups, however the T2 group showed some improvement in intake and significantly increased ADG. In other words, the fact that the total apparent digestibility of nutrients did not improve does not necessarily mean that there was no effect on growth performance. Evaluation of nutrient digestibility depends not only on the total apparent digestibility, but also on the fermentation and retention times of the digesta in the rumen, absorption in the small intestine, and fermentation in the large intestine (30). In the present study, we found an increase in DMI in the T2 group, and it was assumed that supplementation with NSPases had a positive effect on the fiber degradation properties. As shown in a previous study (31), supplementation of dairy cattle rations with enzymes reduced retention time in the rumen and increased the rate of food transfer, thus increasing feed intake but not total intestinal digestibility. This was also studied by Martins et al. (11).

Supplementation with NSPases may increase the availability of the nutrients required for rumen fermentation (11, 32). In the current study, supplementation with NSPases significantly increased the ratio of acetate to propionate, mainly because of the reduced propionate concentration. Ribeiro found that the use of fibrolytic enzymes in sheep diet was consistent with the findings of this study regarding a low propionate concentration and a high acetate to propionate ratio (33). Consistent results were obtained by Miorin et al. (34) in Nellore bulls fed a diet supplemented with xylanase. With the addition of an appropriate amount of NSPases, the enzymes may function synergistically with rumen microbes, preferentially acting on structural carbohydrates and promoting the degradation of non-starch polysaccharides in the rumen (35). It did not cause the accumulation of production or negative feedback effects as in the high dose group. In addition, Nellore bulls fed a high carbohydrate ration supplemented with fibrinolytic enzymes exhibited higher rumen pH, indicating that fibrinolytic enzymes can act during high carbohydrate loading (34). This may be due to changes in microbial populations or metabolic pathways (33). *Prevotella* and *Ruminococcus* are important fiber-degrading genera in the rumen (36). *Prevotella* is a protein-degrading bacterium (37). In our study, the abundances of *Prevotella* and *Ruminococcus* were higher in the T2 group than in the CON group, and the NH_3_-N concentration was significantly higher in the T2 group than in the CON group, presumably indirectly promoting the degradation of dietary proteins and non-starch polysaccharides by changing the abundance of related microbes. Consistent results were also obtained by Guo et al. (38). Thus, in the present study, supplementation with a medium-dose of NSP improved productive performance probably by altering the composition of the sheep rumen microbial community.

Supplementation of fattening sheep diets with high doses of NSPases significantly reduced ADG and ruminal pH, significantly increased feed conversion rate, and exhibited a quadratic curve effect of the enzyme. Similar results have been reported in other studies (3), where excess enzymes attached to the feed competed with the rumen microbial binding sites for feed pellets, thus affecting feed utilization (39, 40). It has also been reported that a negative feedback mechanism occurs when the enzyme-substrate interaction reaches a critical concentration that inhibits enzyme action (41, 42). In this study, a high percentage of concentrate resulted in rapid degradation in the rumen (43). After supplementation with high doses of NSPases, rumen fermentation was further accelerated, which tended to cause accumulation of VFAs, thus inhibiting further fermentation. Therefore, in the present study, the second view was preferred owing to the quadratic curve effect of the enzyme.

As discussed, rumen fermentation was enhanced by supplementation with exogenous NSPases, resulting in a lower pH. In the present study, the enzyme treatment tended to increase rumen NH_3_-N concentration, which is in contrary to the findings of Vittorazzi et al. (10), because the present experiment was conducted after 2 h of morning feeding for rumen fluid collection, which may be related to the fermentation rate. Fiber-degrading bacteria in the rumen have a high preference for ammonia as the nitrogen source (44). In addition, the increased abundance of fiber- and protein-degrading bacteria in this experiment resulted in an increase in the overall utilization of NH_3_-N for MCP in the rumen.

When exogenous enzymes improve rumen fiber digestion, changes in rumen fermentation patterns may occur, resulting in an increase in the molar proportion of acetate and decrease in the molar proportion of propionate (45). Similarly, Ribeiro reported an increase in the total VFA concentration and proportion of acetic acid *in vitro* and a reduced proportion of propionic acid following enzyme treatment (46). In this experiment, acetate was elevated and propionate was decreased in the T2 group, but no significant changes in the VFA profile were observed, which is consistent with the lack of an effect on total digestive tract digestibility, suggesting that supplementation with exogenous NAPases has a minor effect on rumen fiber digestion.

As a significant increase in ADG was observed, we measured the serum hormones levels in each treatment group. GH, secreted by the anterior pituitary gland, is a main regulator of body growth and can be secretes IGF-1 through the liver (47). Animal growth is influenced by the growth hormone GH-IGF1 symbiont axis, in which GH is a major regulator of development, growth, and anabolic processes. IGF1 regulates the biological actions of GH by binding to its receptor (IGF1R) (48).

In the present study, the concentrations of GH and IGF-1 significantly increased after supplementation with NSPases, suggesting that NSPase supplementation promotes an increase in ADG levels, probably by regulating the growth hormone-releasing hormone (HPGH) axis. However, the specific mechanisms and interrelationships among these effects require further investigation. However, nutrient intake and end body weight did not significantly improve in this study. As discussed earlier, the addition of enzymes accelerated the rate of feed degradation in the rumen (49), produced more available energy, and contributed to an increase in associated hormones, but did not increase the extent of feed degradation. Therefore, there is a possibility that the increase in retention time of fiber in the rumen may have counteracted the improvement in digestibility and was not sufficient to cause any improvement in intake and end body weight.

However, the data showed that the T2 group (0.3% NSPases/kg DM) increased 108 g/d, 104.87 g/d, 1.59 g/d, 3.64 g/d, and 0.9 g/d in DM, OM, CP, NDF, and ADF intake, respectively, and all of the test groups were higher than the control group. Therefore, it was speculated that it might be due to the large within-group difference that caused insignificant differences between the results of the test group and the control group. Furthermore, a meta-analysis of studies suggested that long-term serial experiments are preferable for observing the response of exogenous cellulose-degrading enzymes to dietary treatment (6). In this experiment, only a 42 d test cycle was conducted, and the possible effects on growth performance were not demonstrated. Therefore, future subsequent related studies should increase the sample size, reduce within-group variations, and conduct consecutive long-term test cycles.

Leptin is a hormone secreted by white adipose tissue that crosses the blood–brain barrier and affects host appetite and energy metabolism (50–53). It has been shown that short-chain fatty acids (SCFAs), derived from the gastrointestinal tract, enhance the release of leptin and inhibit host appetite (54). There are various pathways for the regulation of related hormones by gastrointestinal flora; for example, derived short-chain fatty acids not only regulate the release of appetite hormones related to the gastrointestinal tract and affect gastrointestinal nerve signaling, but also affect the function and integrity of the intestinal barrier (55–58). In the present study, leptin concentrations gradually increased significantly with increasing NSPases supplementation. As females have a more pronounced maturation rate than males, as well as a greater accumulation of body fat, the consumptive capacity of prehensile females decreases with increasing body weight, as fat directly affects the physical restriction of abdominal fat to the rumen, and indirectly affects food intake through the secretion of leptin by the adipocytes, which is precisely the hormone associated with reduced feeding (59). However, there were no effects until the end of the experimental period, probably because the females had not yet reached sufficient body fat content to trigger a reduction in feed intake. In this study, leptin concentration increased significantly (*p* < 0.05) with the amount of enzyme added, but feed intake was not affected, suggesting that enzyme supplementation during fattening did not negatively affect the experimental sheep, or that the negative effects were not manifested, possibly because sufficient body fat content had not yet been achieved to trigger a reduction in feed intake.

Because the medium-dose group showed better results, we measured rumen microbe in the CON and T2 groups. In the present study, the bacterial abundance increased in the T2 group, in which *Firmicutes* and *Bacteroidota* were the dominant phylum in the rumen of sheep, consistent with the results of previous studies (60, 61). Among them, the *Bacteroidota* was mainly for the energy conversion and acquisition, and the *Firmicutes* mainly played an essential role in the degradation of non-structural carbohydrates (62, 63). Both the *Bacteroidota* and *Firmicutes* were more abundant in the T2 group than in the CON group. This indicates that supplementation with NSPases promotes non-structural carbohydrate degradation and energy uptake. Another study reported that feeding high concentration resulted in higher abundance of *Firmicutes* in the rumen than feeding on high-fiber diets (64). In the present study, supplementation with NSPases resulted in a higher abundance of the *Firmicutes* in the T2 group than in the CON group, confirming that supplementation with NSPases increased the rate of degradation and energy conversion in the rumen and creating the illusion that the T2 group had a higher non-structural carbohydrate content than the CON group.

Interestingly, we found significantly lower abundances of *Uroviricota* and *Proteobacteria* in the T2 group than in the CON group. When the number of *Proteobacteria* is low, it behaves benignly; when it is more abundant, it may become a microbe that triggers inflammation, and its long-term enrichment may represent microbial instability or disease state (65). Some *Proteobacteria* are easily degraded and produce metabolites after death capable of destroy the mucosal barrier in the gastrointestinal tract (65). In contrast, *Bifidobacteria,* a major genus of *Actinobacteria* can avoid excessive inflammation (66). It has also been reported that the maintenance of gastrointestinal homeostasis depends on (SCFAs) as microbial metabolic byproducts (e.g., acetate, propionate, and butyrate) and that these acids affect immune responses in the intestine and other organs and tissues (67, 68). The regulatory relationship between microbial-derived fatty acids and host defense peptides was elaborated. In the present study, it was found that supplementation with NSPases not only increased the abundance of the *Bacteroidota*, *Firmicutes* and *Actinobacteria*, but also resulted in a significant increase in immunoglobulins, accompanied by a decrease in the abundance of viruses in rumen microbes, suggesting that the addition of appropriate amounts of NSPases to high concentrate rations can lead to antiviral effects and help improve rumen health, which may be associated with altered rumen microbes.

Among them, acetate concentration had a significant positive correlation with *Prevotella* and propionate had a significant positive correlation with *unclassified_f_Lachnospiraceae*, and both *Prevotella* and *unclassified_f_Lachnospiraceae* abundance were higher in the T2 group than in the CON group, indicating that the potential of acetate and propionate was higher in the T2 group than in the CON group, but in reality the propionate concentration was lower in the T2 group than in the CON group, presumably because the supplementation of NSPases improved in rumen health and helped rumen propionate fermentation, which led to faster uptake of propionic acid by the rumen epithelium, resulting in low measured concentrations. Moreover, propionate, as a precursor of gluconeogenesis, can be absorbed by the liver for use by the organism, ultimately leading to an increase in ADG in the T2 group. This also explains why total apparent digestibility was not improved by NSPase supplementation, while the ADG was improved. Therefore, NSPases supplementation improves rumen health, promotes rumen fermentation, and enhanced energy absorption and utilization. Previous studies have shown that enzyme preparations based on *Aspergillus* can increase bacterial populations (69, 70) an act synergistically with rumen microbes to break down plant cell walls and promote nutrient release (71).

Using the Wilcoxon rank-sum test of the abundance of CAZy in the CON and T2 groups, we found that with NSPases supplementation promoted the degradation of NSP, which also confirmed that NSPases supplementation helped improve rumen fermentation after feeding high concentrates.

## 5 Conclusion

In conclusion, supplementation with non-starch polysaccharide degrading enzymes increased average daily weight gain, improved rumen health, and enhanced immunity. Feeding with NSPases improves rumen health and promotes energy absorption and utilization by increasing immunoglobulin concentrations and decreasing rumen viral abundance.

## Data availability statement

The metagenome sequences obtained from sheep rumen fluids have been deposited in the NCBI Short Read Archive (GenBank accession number: PRJNA978373).

## Ethics statement

The animal studies were approved by Approval Letter of Biology Ethics Committee of Shihezi University. The studies were conducted in accordance with the local legislation and institutional requirements. Written informed consent was obtained from the owners for the participation of their animals in this study.

## Author contributions

YX: Writing – original draft, Writing – review & editing. HS: Writing – review & editing. HG: Validation, Writing – review & editing. CN: Project administration, Writing – review & editing. SN: Writing – review & editing. QL: Resources, Writing – review & editing. CC: Writing – review & editing. WZ: Writing – review & editing.

## Footnotes

^1^ https://github.com/OpenGene/fastp

^2^ https://github.com/bbuchfink/diamond
