# Supplementary material for: Effect of the supplementation of exogenous complex non-starch polysaccharidases on the growth performance, rumen fermentation and microflora of fattening sheep
Source: Front Vet Sci. 2024 May 16;11:1396993. doi: 10.3389/fvets.2024.1396993 (PMC11138346; doi:10.3389/fvets.2024.1396993)
Supplement: Supplementary file 1 [file Data_Sheet_1.docx]

**Effect of the supplementation of exogenous complex non-starch polysaccharidases on the growth performance, rumen fermentation and microflora of fattening sheep**

**Yuyang Xue** ^1^, **Haobin Sun** ^1^, **Hongyong Guo** ^2^, **Cunxi Nie** ^1^, **Shanshan Nan** ^1^, **Qicheng Lu** ^1^, **Cheng Chen** ^1, *^, **Wenju Zhang** ^1, *^

Supplementary Table S1

Effect of NSPases supplementation on Kingdom-level annotations for rumen samples of sheep.

| Taxon | Treatment^1^ (% composition) | | SEM | *P*-Value |
| --- | --- | --- | --- | --- |
|  | CON | T2 |  |  |
| Bacteria | 81.24 | 89.27 | 2.89 | 0.175 |
| Eukaryota | 0.41 | 0.17 | 0.13 | 0.386 |
| Archaea | 5.29 | 1.92 | 0.99 | 0.091 |
| Viruses | 13.04 | 8.63 | 2.37 | 0.377 |
| NA | 0.017 | 0.012 | 0.002 | 0.376 |

Abbreviations: NA = not annotated.

^1^ Different supplementary level (CON, control group; T2, 0.3% NSPases/kg DMI).

Supplementary Table S2

Effect of NSPases supplementation on Phylum-level annotations for rumen samples of sheep.

| Phylum | Treatments^1^ (% composition) | | SEM | P-value |
| --- | --- | --- | --- | --- |
|  | CON | T2 |  |  |
| Firmicutes | 48.14 | 52.91 | 4.29 | 0.602 |
| Bacteroidota | 12.68 | 16.98 | 2.50 | 0.415 |
| Actinobacteria | 8.78 | 14.42 | 2.57 | 0.294 |
| Uroviricota | 12.50^a^ | 5.43^b^ | 1.67 | 0.026 |
| Proteobacteria | 7.31^a^ | 3.10^b^ | 1.06 | 0.040 |
| Euryarchaeota | 4.61 | 1.87 | 0.88 | 0.122 |

^1^ Different supplementary level (CON, control group; T2, 0.3% NSPases/kg DMI).

^a-c^ Values within a row with different superscripts differ significantly at P < 0.05.

Supplementary Table S3

Effect of NSPases supplementation on Genus-level annotations for rumen samples of sheep.

| Genus | Treatment^1^ (%composition) | | SEM | *P*-value |
| --- | --- | --- | --- | --- |
|  | CON | T2 |  |  |
| unclassified_f__Lachnospiraceae | 13.05 | 14.98 | 1.81 | 0.619 |
| Prevotella | 5.06 | 7.77 | 1.57 | 0.413 |
| unclassified_c__Clostridia | 5.61 | 6.36 | 0.78 | 0.651 |
| Ruminococcus | 2.59 | 6.41 | 2.49 | 0.471 |
| Clostridium | 4.32 | 3.48 | 1.45 | 0.789 |
| unclassified_f__Siphoviridae | 5.41 | 2.41 | 0.09 | 0.097 |
| Aeriscardovia | 0.53 | 6.16 | 2.99 | 0.371 |
| unclassified_o__Bacteroidales | 3.41 | 3.74 | 0.94 | 0.869 |

^1^ Different supplementary level (CON, control group; T2, 0.3% NSPases/kg DMI).
